# Mechanism of Bacterial Inactivation by (+)-Limonene and Its Potential Use in Food Preservation Combined Processes

**DOI:** 10.1371/journal.pone.0056769

**Published:** 2013-02-12

**Authors:** Laura Espina, Tilahun K. Gelaw, Sílvia de Lamo-Castellví, Rafael Pagán, Diego García-Gonzalo

**Affiliations:** 1 Departamento de Producción Animal y Ciencia de los Alimentos, Facultad de Veterinaria, Universidad de Zaragoza, Zaragoza, Spain; 2 Departament d'Enginyeria Química, Universitat Rovira i Virgili, Avinguda Païssos Catalans, Tarragona, Spain; Universidad Nacional de La Plata., Argentina

## Abstract

This work explores the bactericidal effect of (+)-limonene, the major constituent of citrus fruits' essential oils, against *E. coli*. The degree of *E. coli* BJ4 inactivation achieved by (+)-limonene was influenced by the pH of the treatment medium, being more bactericidal at pH 4.0 than at pH 7.0. Deletion of *rpoS* and exposure to a sub-lethal heat or an acid shock did not modify *E. coli* BJ4 resistance to (+)-limonene. However, exposure to a sub-lethal cold shock decreased its resistance to (+)-limonene. Although no sub-lethal injury was detected in the cell envelopes after exposure to (+)-limonene by the selective-plating technique, the uptake of propidium iodide by inactivated *E. coli* BJ4 cells pointed out these structures as important targets in the mechanism of action. Attenuated Total Reflectance Infrared Microspectroscopy (ATR-IRMS) allowed identification of altered *E. coli* BJ4 structures after (+)-limonene treatments as a function of the treatment pH: β-sheet proteins at pH 4.0 and phosphodiester bonds at pH 7.0. The increased sensitivity to (+)-limonene observed at pH 4.0 in an *E. coli* MC4100 *lptD4213* mutant with an increased outer membrane permeability along with the identification of altered β-sheet proteins by ATR-IRMS indicated the importance of this structure in the mechanism of action of (+)-limonene. The study of mechanism of inactivation by (+)-limonene led to the design of a synergistic combined process with heat for the inactivation of the pathogen *E. coli* O157:H7 in fruit juices. These results show the potential of (+)-limonene in food preservation, either acting alone or in combination with lethal heat treatments.

## Introduction

The compound (+)-limonene is the major constituent of citrus fruits' essential oils (EOs) [1], [2]. Because of its citrus-like flavor, (+)-limonene is employed as a flavoring agent in perfumes, creams, soaps, household cleaning products and in some food products such as fruit beverages and ice creams [3]. In addition, (+)-limonene has been found to possess antifungal [4], [5], bacteriostatic [6], [7] and bactericidal [8] properties. Therefore, its use as a food preservative has also been proposed [9].

Antimicrobial compounds have been successfully combined with other preservation technologies in order to achieve a synergistic effect in the inactivation of the target pathogens, following the hurdle theory proposed by Leistner and Gorris [10]. For example, exposure of *Escherichia coli* or *Cronobacter sakazakii* to citral combined with high hydrostatic pressure (HHP), pulsed electric fields (PEF) or heat treatments, respectively, increased the inactivation degree achieved for each hurdle acting alone [11], [12], [13]. Similarly, plenty of other compounds present in EOs were found to be effective in combination with heat in the inactivation of *E. coli* and *Listeria monocytogenes* [14]. Combinations of (+)-limonene with heat or non-thermal technologies could likewise yield a similar synergistic effect in the inactivation of the target pathogens while preserving the organoleptic properties of the fresh food product.

The use of (+)-limonene in the design of food preservation processes requires a proper understanding of its mechanism of inactivation and of the influence of environmental factors that might affect it. (+)-Limonene belongs to the cyclic monoterpene hydrocarbon family, which are considered to accumulate in the microbial plasma membrane and thus cause a loss of membrane integrity and dissipation of the proton motive force [15]. Previous studies on the inactivation of *E. coli* by other terpenes and terpenoids (such as carvacrol or citral) have demonstrated the occurrence of sub-lethal injury in the outer and cytoplasmic membranes [13], [14], pointing out the membrane disruption as a mechanism of inactivation by these compounds. However, the precise targets of terpenes and terpenoids are not yet completely understood.

Description of cellular target of antimicrobial compounds could be assisted by the use of Fourier transform-infrared (FT-IR) spectroscopy [16]. FT-IR spectroscopy is a physico–chemical analytical technique based on measurement of vibration of a molecule excited by IR radiation at a specific wavelength range. Specially, attenuated total reflectance infrared microspectroscopy (ATR-IRMS) provides bands from all the cellular components of microorganisms (e.g. cell membrane and wall components, proteins and nucleic acids), giving spectral signatures or “fingerprints” that permit the classification of a microorganism at the strain and serovar level [17].

Regulation of gene expression by alternative sigma factors, which are proteins that act as transcription initiation factors through specific binding of RNA polymerase to gene promoters is key in bacterial resistance to food preservation technologies [18]. In many Gram-negative and Gram-positive genera, sigma factors, σ^S^ (encoded by *rpoS* gene) and σ^B^ (encoded by *sigB* gene), respectively, are responsible for the transcription of specific stationary-phase genes [19]. Besides, these sigma factors could also be responsible for cell protection under environmental stresses such as acid, cold, heat or osmotic shocks [19], [20], [21]. Since previous work showed that the expression of RpoS contributed to the higher resistance of *E. coli* to the terpene aldehyde citral [13], a similar regulation could be expected for other chemical compounds such as (+)-limonene.

The aims of this work were: (a) to study the inactivation of *Escherichia coli* BJ4 by (+)-limonene, describing the effect of the pH of the treatment medium, deletion of sigma factor σ^S^ and sub-lethal shocks; (b) to study the occurrence of lethal and sub-lethal injuries caused by (+)-limonene in bacterial envelopes of *E. coli* BJ4 and MC4100; (c) to identify the *E. coli* BJ4 structures affected by (+)-limonene through ATR-IRMS spectroscopy, and (d) to determine the synergistic lethal effect obtained when combining (+)-limonene with heat and PEF treatments to inactivate *E. coli* O157:H7.

To accomplish these objectives we used different *E. coli* strains. In the first part, dedicated to describing the mechanism of inactivation by (+)-limonene the strains *E. coli* BJ4 and its Δ*rpoS* mutant [22] were used to study the influence of this alternative sigma factor in the bacterial resistance to (+)-limonene, and *E. coli* K-12 MC4100 and its Δ*lptD4213* mutant [23] to study the role of the outer membrane in this resistance. In the second part, dedicated to demonstrating that knowledge of the mechanism of inactivation by (+)-limonene may have an applied interest to develop food preservation combined processes. Thus, we evaluated the efficacy of a combined process using (+)-limonene to inactivate the foodborne bacterium *E. coli* O157:H7 in fruit juices in which this pathogen uses to cause food safety problems.

## Materials and Methods

### Micro-organisms and growth conditions

The strains used were *Escherichia coli* BJ4 and its Δ*rpoS* null mutant BJ4L1 [22], *E. coli* K-12 MC4100 and its Δ*lptD4213* mutant [23] and *E. coli* O157:H7 VTEC - (Phage type 34) [24]. The cultures were maintained in cryovials at -80 °C prior to use. Broth subcultures were prepared by inoculating one single colony from a plate, a test tube containing 5 mL of sterile tryptic soy broth (Biolife, Milan, Italy) with 0.6% yeast extract added (Biolife) (TSBYE). After inoculation, the tubes were incubated overnight at 37 °C. With these subcultures, 250 mL Erlenmeyer flasks containing 50 mL of TSBYE were inoculated to a final concentration of 10^4^ CFU/mL. These flasks were incubated with agitation (130 rpm; Selecta, mod. Rotabit, Barcelona, Spain) at 37° C until the stationary growth phase was reached.

### Bacterial treatment with (+)-limonene

(+)-Limonene (97% purum) was purchased from Sigma-Aldrich (Sigma-Aldrich Chemie, Steinheim, Germany). This compound is practically immiscible in water, so a vigorous shaking method was used to prepare suspensions. (+)-Limonene was added at a final concentration of 200 µL/L to tubes containing 10 mL of citrate-phosphate buffer of pH 4.0 (23.85 g/L) and 7.0 (27.09 g/L). Before treatments, bacterial cultures were centrifuged at 6,000•*g* for 5 min and resuspended in the same buffer that of each treatment. Microorganisms were added at a final concentration of 3·10^7^ CFU/mL and maintained under constant agitation (130 rpm) at 20 °C. Samples were taken at regular intervals, and survivors were enumerated. According to previous studies [13], [14], treatment time and temperature; and initial concentrations of (+)-limonene and bacteria were chosen to detect 5 log_10_ cycles of cell inactivation (i.e. from 3·10^7^ to 3·10^2^ CFU/mL). Minimal inhibitory concentration (MIC) of (+)-limonene determined using the tube dilution method [25] for *E. coli* BJ4 and O157:H7 was 5 µL/mL (data not shown).

Previous experiments showed that native *E. coli* was insensitive to incubation in citrate–phosphate buffer at pH 7.0 or pH 4.0 for 24 h at 20 °C.

### Sub-lethal heat, cold and acid shock treatments

One 1-mL aliquot of bacterial suspensions was centrifuged at 10,000•*g* for 5 min and resuspended in 1 mL of TSBYE at 45°C or 0°C (sub-lethal heat and cold shocks, respectively) or in TSBYE acidified to pH 4.5 with HCl at 20 °C (sub-lethal acid shock). Sub-lethal heat shock was performed by immersing the bacterial suspensions in a thermostatic water bath (Bunsen, mod. BTG, Madrid, Spain) and holding at 45 °C for 2 h. Suspensions were kept on ice for 4.5 h (sub-lethal cold shock) or at 20°C (sub-lethal acid shock) for 1 h. Microbial resistance to (+)-limonene was assessed as explained above. These conditions were chosen from previous published work [26], [27].

### Cell permeabilization by (+)-limonene

Permanent cell permeabilization of *E. coli* BJ4 was evaluated after the treatment with 200 µL/L of (+)-limonene (initial cell concentration: 3·10^7^ CFU/mL) for different treatment times (10 min, 25 min, 1 h, 6 h, 24 h) at pH 4.0 and 7.0 at 20° C. Cells were centrifuged, supernatant was removed, and propidium iodide (PI) (Sigma – Aldrich, Madrid, Spain) was added to a final concentration of 0.08 mmol/L [28]. Cell suspensions were incubated for 15 min at 20° C, centrifuged at 10,000·g for 5 min, and washed three times until no extracellular PI remained in the buffer. Cell permeabilization was analyzed using a fluorescence microscope (Nikon, Mod. L-Kc, Nippon Kogaku KK, Japan).

### Attenuated total reflectance infrared microspectroscopy (ATR-IRMS) with multivariate analysis

An aliquot of cell suspensions was centrifuged at 6,000·*g* for 10 min at 4° C. Pellets were washed three times with 1 mL of 0.9% NaCl and centrifuged at 6,000·*g* for 10 min. Pellets were placed onto grids of hydrophobic membrane (HGM; ISO-GRID, Neogen Corporation, Lansing, MI, USA) and dried out under laminar flow at room temperature for 1 h. Samples were analyzed by IR equipment (Illuminate IR, Smiths detection, The Genesis Centre Science Park South Birchwood Warrington, United Kingdom) interfaced with mercury-cadmium-telluride photoconductive detector and equipped with a microscope with a motorized x-y stage, 20x and 50x objectives, and slide-on attenuated total reflection (ATR) diamond objective (Smiths detection, United Kingdom). The hydrophobic membranes were placed on the stage of the microscope and a specific position of the microbial pellet was selected with the assistance of the microscope and live camera (Leica OM 2,500, Module FT-IR, Renishaw plc, New Mills, Wotton-under-Edge, Gloucestershire, United Kingdom). The microscope was software-controlled using Wire 3.2 version software (Renishaw plc, United Kingdom). Spectra were collected from 4,000 to 800 cm^−1^ with a resolution of 4 cm^−1^. The spectrum of each sample was obtained by taking the average of 128 scans. Spectra were displayed in terms of absorbance obtained by rationing the single beam spectrum against that of the air background. The spectrometer was completely software controlled by synchronize IR basic version 1.1 software (SensIR Technologies, Smiths detection, United Kingdom). Pirouette® multivariate analysis software (version 4.0, InfoMetrix, Inc., Woodville, WA) was used to analyze the raw spectra of bacterial cells. The IR spectral data were mean-centred, transformed to their second derivative using a 15-point Savitzky-Golay polynomial filter, and vector-length normalized; sample residuals and Mahalanobis distance were used to determine outliers [29], [30]. Soft independent modeling of class analogy (SIMCA) was used to build a predictive model based on the construction of separate principal component analysis (PCA) models for each class. SIMCA class models were interpreted based on class projections, misclassifications and discriminating power. Class projections were visible through three-dimensional graphics of clustered samples. Probability clouds (95%) are built around the clusters based on PCA scores, allowing SIMCA to be used as a predictive modeling system. Total misclassifications were analyzed and interpreted for the input data. Variable importance, also known as discriminating power, was used to define the variables (wavenumbers) that have a predominant effect on bacterial classification, minimizing the difference between samples within a cluster, and maximizing differences between samples from different clusters. SIMCA analysis assesses itself by predicting each sample included in the training set comparing that prediction to its assigned class; this assessment is referred to as misclassifications. Zero misclassifications typify a model in which all samples were correctly predicted to the pre-assigned class. Compared samples were *E. coli* BJ4 (initial concentration: 3·10^7^ CFU/mL) after being incubated for 24 h at 20° C in absence or presence of 200 µL/L of (+)-limonene in citrate-phosphate buffer of pH 4.0 or 7.0.

### Duration of lag phase in untreated and (+)-limonene-treated cells

*E. coli* BJ4 at an initial concentration of 3·10^7^ CFU/mL were treated for 20 min with 200 µL/L of (+)-limonene in citrate – phosphate buffer of pH 4.0 so that 1 log_10_ cycle of inactivation was reached. At this moment, cells were centrifuged and resuspended in TSBYE without (+)-limonene. Non-treated cells were also centrifuged, resuspended in TSBYE without (+)-limonene and adjusted at the same final concentration of live cells (3·10^6^ CFU/mL). Optical absorbance was measured at 590 nm during growth for 14 h of both samples at 37 °C with a spectrophotometer (GENios, Tecan, Austria).

### Microbial inactivation by the combination of a lethal heat treatment and (+)-limonene

Tubes containing 5 mL of apple juice or orange juice in absence or presence of (+)-limonene added to a final concentration of 200 µL/L were placed in a shaking thermostatic bath at 54 °C (Bunsen, mod. BTG, Madrid, Spain). Before treatments, bacterial suspensions of *E. coli* O157:H7 were centrifuged at 10,000·*g* for 5 min and resuspended in apple or orange juice. Once the treatment temperature was reached, the microbial suspension was added to a final concentration of 3·10^7^ CFU/mL. Samples were taken after 10 min and survivors were enumerated. These treatment conditions were chosen to make these data comparable with previously published data obtained under the same conditions [1], [12], [14], [31].

### Microbial inactivation by the combination of pulsed electric fields and (+)-limonene

PEF treatments were carried out in an equipment that delivered an exponential-decay pulse previously described by García et al. [32], provided with a parallel-electrode treatment chamber with a distance of 0.25 cm between electrodes and an area of 2.01 cm^2^. Before treatments, bacterial suspensions of *E. coli* O157:H7 were centrifuged at 10,000·*g* for 5 min and resuspended in shelf-stable apple juice (pH 3.6) or orange juice (pH 3.8) (Don Simón, Murcia, Spain). Bacterial cultures were added to tubes containing 5 mL of each of these media with or without 200 µL/L of (+)-limonene, and 0.5 mL of these suspensions were placed into the treatment chamber with a sterile syringe. Exponential waveform pulses at an electrical field strength of 30 kV/cm and a pulse repetition rate of 1 Hz were used in this study. Experiments started at room temperature and after the application of 25 pulses the temperature of the samples was below 35° C. After treatment, samples were taken and survivors were evaluated.

### Survival counts

After treatments, samples were adequately diluted in 0.1% w/v Peptone Water (Biolife), containing 1% v/v Tween 80 (Biolife) as a neutralizer. 0.1 ml aliquots from the neutralized samples were pour-plated onto Tryptic Soy Agar with 0.6% Yeast Extract added (TSAYE) as non-selective medium. Plates were incubated at 37°C for 24 h. Previous experiments showed that longer incubation times did not influence the survival counts. In order to determine bacterial cell injury, treated samples were also plated onto selective media: TSAYE with 4% (*E. coli* BJ4 and MC4100) or 3% (*E. coli* BJ4L1, O157:H7 and MC4100 Δ*lptD4213*) sodium chloride (Sigma-Aldrich, Madrid, Spain) added (TSAYE-SC) to evaluate cytoplasmic membrane damage; and onto TSAYE with 0.35% (*E. coli* O157:H7) or 0.2% (*E. coli* BJ4 and BJ4L1) bile salts (Biolife) added (TSAYE-BS) to evaluate outer membrane damage. These levels of sodium chloride and bile salts were determined as the maximum non-inhibitory concentrations for native cells. Plates containing selective media were incubated for 48 h at 37°C. Previous experiments showed that longer incubation times did not influence survival counts.

After incubation of plates, colonies were counted with an improved image analyzer automatic counter (Protos; Analytical Measuring Systems, Cambridge, United Kingdom) as described by Condón et al. [33]. The extent of sub-lethal injury was expressed as the difference between the log_10_ count (CFU) on non-selective medium (TSAYE) and the log_10_ count on selective media (TSAYE-SC and TSAYE-BS) after treatments. According to this representation, “2 log_10_ cycles of injured cells” means a 2-log_10_ difference in the count on selective and non-selective media or that 99% of survivors were sub-lethally injured.

### Statistical analysis

Experiments were carried out in triplicate on different working days. Inactivation was expressed in terms of the extent of reduction in log_10_ counts after every treatment. The error bars in the figures indicate the mean ± standard error from the data obtained from at least three independent experiments. Analyses of variance (*p* = 0.05) were performed using SPSS software (SPSS, Chicago, IL, USA).

To characterize the growth kinetics, the absorbance values were fit using nonlinear regression with the Gompertz model [34], which in this case can be described by the equation:
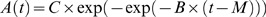
where *A(t)* is the absorbance value in time *t*, *C* is the absorbance value in the stationary phase, *B* is the relative growth rate in point *M*, and *M* is the time in which the cells reach their maximum growth rate. The lag phase duration was calculated as 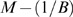.

## Results

### Inactivation of *Escherichia coli* BJ4 by (+)-limonene: effect of treatment medium pH, deletion of *rpoS* and sub-lethal shocks

**Figure 1:**
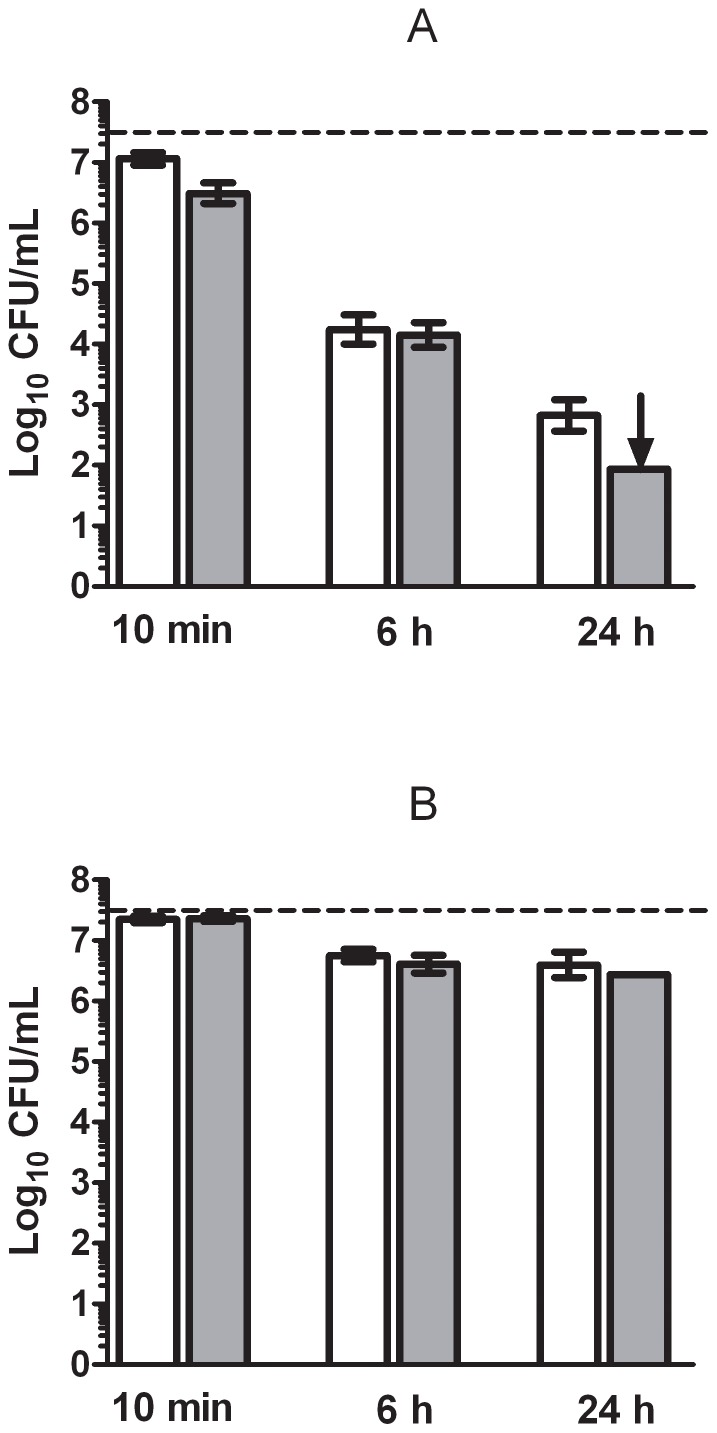
Study of the effect of time, pH and *rpoS* deletion on *Escherichia coli* BJ4 inactivation by (+)-limonene. Log_10_ of survival counts of *Escherichia coli* BJ4 (wild type: □) and BJ4L1 (Δ*rpoS*: 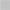) after 10 min, 6 h and 24 h with 200 µL/L of (+)-limonene in citrate-phosphate buffer of pH 4.0 (A) or 7.0 (B) at 20° C. Cells were recovered in TSAYE. Discontinuous line indicates initial cell concentration (3·10^7^ CFU/mL). Arrow indicates survival counts under detection limit. Error bars indicate standard error.

The antimicrobial activity of 200 µL/L of (+)-limonene on the survival of 3·10^7^ CFU/ml of *E. coli* BJ4 and its *rpoS* mutant BJ4L1 was tested at pH 4.0 and 7.0 for 10 min, 6 h and 24 h (Figure 1). Both *E. coli* strains were less resistant at pH 4.0 than at pH 7.0: after 24 h of treatment less than 2 log_10_ cycles of the initial populations were inactivated at pH 7.0 (Figure 1B), while a treatment of 6 h at pH 4.0 was able to inactivate more than 3 log_10_ cycles of both strains, and about 5 log_10_ cycles of inactivation were achieved after 24 h of storage (Figure 1A).

Regarding the comparison between the wild and the mutant strain, both wild type and *rpoS* mutants showed a similar (+)-limonene resistance for all the treatments assayed (*p*>0.05).

Development of cross-resistance to (+)-limonene as a consequence of sub-lethal shocks was studied. On the one hand, exposure to a previous sub-lethal heat or acid shock did not affect the final inactivation reached by (+)-limonene in *E. coli*, since no statistically significant differences were found when compared to the control treatment (*p*>0.05). On the other hand, exposure to a sub-lethal cold shock significantly decreased (*p*<0.05) the resistance of both strains to (+)-limonene (Table 1).

**Table 1:** Influence of previous sub-lethal heat, cold and acid treatments on *Escherichia coli* BJ4 resistance to (+)-limonene.

	*Escherichia coli* BJ4		*Escherichia coli* BJ4L1
Control	4.24^a^±0.24	4.16^a^±0.21	
Heat-shock	4.00^a^±0.18	3.72^a^±0.33	
Cold-shock	3.00^b^±0.41	<2.18^c^	
Acid-shock	4.89^a^±0.21	4.55^a^±0.23	

a, b, c : same letters indicate non-significant differences among mean values; *p*>0.05.

Log_10_ of survival counts (CFU/mL) of *Escherichia coli* BJ4 (wild type) and BJ4L1 (Δ*rpoS*) (initial concentration: 3·10^7^ CFU/mL) by a treatment with 200 µL/L of (+)-limonene in citrate-phosphate buffer of pH 4.0. Table includes data from non-stressed cells (control) and cells exposed to different sub-lethal shocks before (+)-limonene treatments (mean ± standard error). Sub-lethal heat shock: 45 °C/2 h; sub-lethal cold shock: ice/4.5 h; sub-lethal acid shock: pH 4.5/1 h.

Bacterial counts were not modified (*p*>0.05) by incubation in citrate–phosphate buffer at pH 7.0 or pH 4.0 without (+)-limonene for 24 h at 20 °C (data not shown).

### Occurrence of sub-lethal damage after (+)-limonene treatments in *E. coli* BJ4

**Figure 2:**
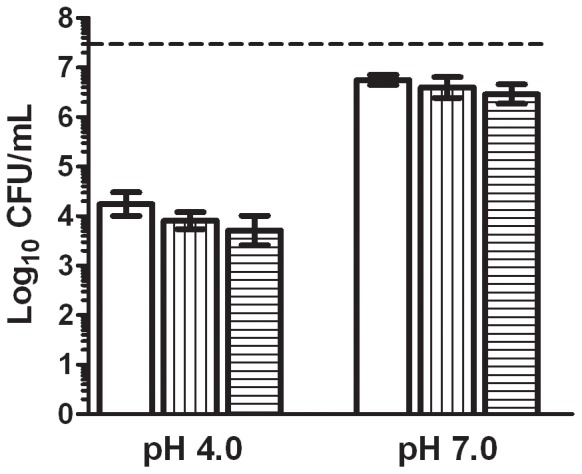
Study of sub-lethal injury caused by (+)-limonene on *Escherichia coli* BJ4 determined using selective plating technique. Log_10_ of survival counts of *Escherichia coli* BJ4 after 6 h with 200 µL/L of (+)-limonene in citrate-phosphate buffer of pH 4.0 or 7.0 at 20° C. Cells were recovered in TSAYE (□), TSAYE-SC (sodium chloride: vertical stripes) or TSAYE-BS (bile salts: horizontal stripes). Discontinuous line indicates initial cell concentration (3·10^7^ CFU/mL). Error bars indicate standard error.

The enumeration of the survivors on the selective medium TSAYE-SC (with sodium chloride) and TSAYE-BS (with bile salts) (Figure 2) revealed that storage for 6 h with 200 µL/L of (+)-limonene caused sub-lethal damages neither to the cytoplasmic nor to the outer membrane of *E. coli* BJ4, since the levels of inactivation in these media were similar to those detected in TSAYE for each pH (*p*>0.05, Fig. 1). The same conclusion was drawn from the survival counts after 10 min and 24 h of treatment (data not shown).

Bacterial counts in selective or non-selective media were not modified (*p*>0.05) by incubation in citrate–phosphate buffer at pH 7.0 or pH 4.0 without (+)-limonene for 24 h at 20 °C (data not shown).

Moreover, we evaluated the growth of treated and non-treated cells after exposure/non-exposure to (+)-limonene. Since exponential phase started after 2 h post-inoculation in both populations (2.19±0.19 h) no lag phase delay was detected in treated cells (*p*>0.05) (data not shown).

### Membrane permeabilization of *E. coli* BJ4 by (+)-limonene

**Figure 3:**
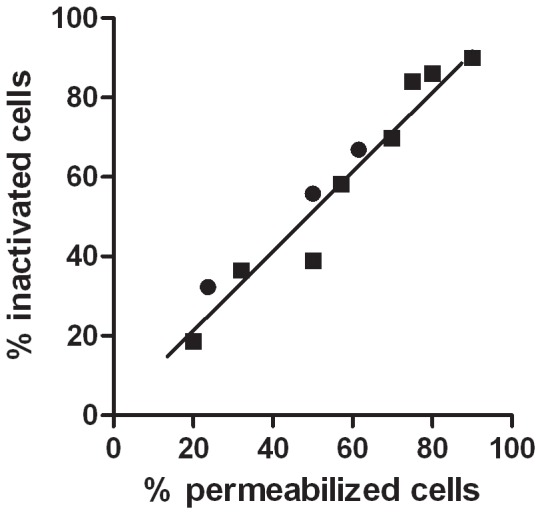
Correlation between permeabilized and inactivated *Escherichia coli* BJ4 cells. Correlation between the percentage of inactivated *Escherichia coli* BJ4 and the percentage of cells stained with propidium iodide (permeabilized cells) after different treatment times (10 min, 25 min, 1 h, 6 h, 24 h) with 200 µL/L of (+)-limonene (initial cell concentration: 3·10^7^ CFU/mL) at pH 4.0 (•) or 7.0 (▪).

Permanent membrane permeabilization of *E. coli* BJ4 was demonstrated by the uptake of the fluorescent probe PI. As can be seen in Figure 3, a direct correlation (*R^2^* = 0.96) was found between the percentage of inactivated cells and the percentage of permeabilized cells after adding 200 µL/L of (+)-limonene for different treatment times. Furthermore, as seen with cell inactivation, the percentage of permeabilized cells after 10 min of exposure to (+)-limonene was influenced by pH: after 1 h, *E. coli* showed maximum cell permeabilization (>90%) at pH 4.0, corresponding to more than 2 log_10_ cycles of cell inactivation, while at pH 7.0 only about 50% of cells were permeabilized and inactivated. After incubation in the presence of (+)-limonene for 24 h, maximum cell permeabilization (>90%) was observed at both pHs.

No membrane permeabilization (*p*>0.05) was detected after incubation in citrate–phosphate buffer at pH 7.0 or pH 4.0 without (+)-limonene for 24 h at 20 °C (data not shown).

### Attenuated total reflectance infrared microspectroscopy of *E. coli* BJ4 cells after (+)-limonene treatments

**Figure 4:**
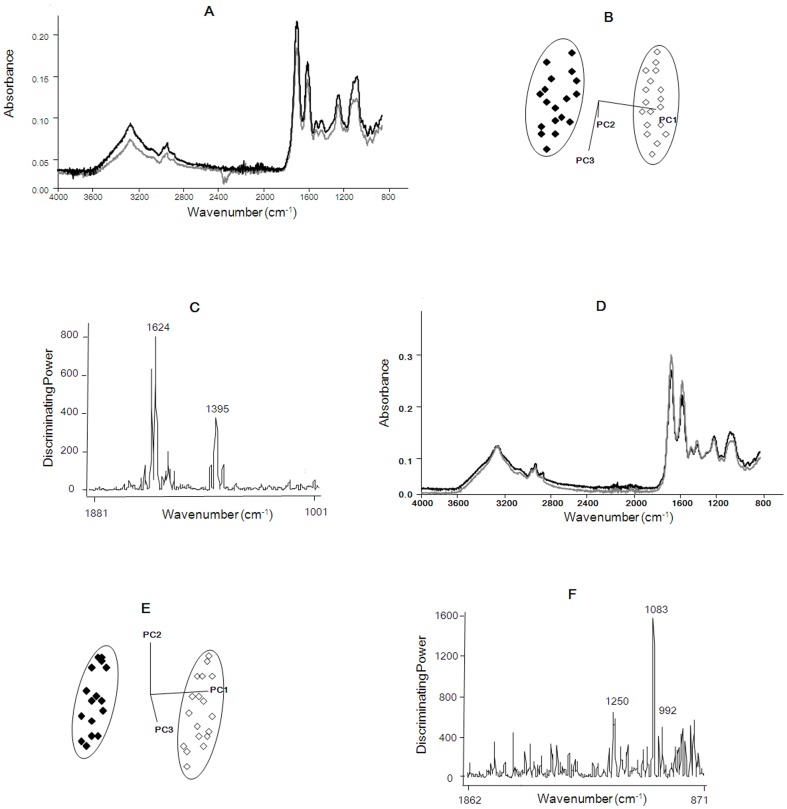
ATR-IRMS spectra of (+)-limonene treated *Escherichia coli* BJ4 cells. Typical raw spectra (A, D) and soft independent modeling class analogy (SIMCA) of class projections (B, E) and discriminating power (C, F) of non-treated and (+)-limonene treated (200 µL/L) *Escherichia coli* BJ4 (initial concentration: 3·10^7^ CFU/mL) at pH 4.0 (A, B, C) or 7.0 (D, E, F) of transformed attenuated total reflectance infrared micro spectroscopy (ATR-IRMS) spectra. Black and gray lines and symbols represent non-treated (+)-limonene treated cells, respectively. Spectra were obtained from three independent samples.

Typical spectra of *E. coli* BJ4 with the presence or absence of (+)-limonene at pH 4.0 and 7.0 are shown in Figures 4A and 4D, respectively. Class projections illustrate the ability of SIMCA to differentiate IR data based on the first 3 principal components. Since the range of 4000 to 2000 cm^−1^ was not significant to describe the biochemical differences among our samples Figures 4B, 4C, 4E and 4F only includes data obtained from the range 1,900–800 cm^−1^. Our classification models obtained from derivatized infrared spectra (1900–800 cm^−1^) of *E. coli* BJ4 cells (Figures 4B and 4E) allowed for the tight clustering and clear differentiation of *E. coli* BJ4 samples according to the presence or absence of (+)-limonene for each pH. Discriminating power of SIMCA is a measure of variable importance in infrared frequency and contributes to the development of the classification model [30]. Figures 4C and 4F show the wavenumbers that had a predominant effect on discrimination of (+)-limonene-treated and untreated cells at pH 4.0 and 7.0, respectively. As can be seen, the discriminating power of non-treated and (+)-limonene treated samples at pH 4.0 (Figure 4C) showed two spectral bands at 1,624 and 1,395 cm^−1^, corresponding to changes in the amide I absorption band of β-sheet proteins [35], [36]; and in the symmetric stretching of COO^-^ groups in amino acids and/or fatty acids [37], [38], [39]. At pH 7.0 (Figure 4F), comparison of (+)-limonene treated and non-treated cells showed that the major discriminating bands were those located at 1,083, 1,250 and 992 cm^−1^, corresponding to the symmetric and asymmetric stretching of P = O groups in phosphodiester bonds and ring vibrations of carbohydrates [16], [37], [40]. ATR-IRMS spectra of (+)-limonene treated *E. coli* O157:H7 allowed us obtaining similar conclusions (data not shown).

### Role of *E. coli* MC4100 outer membrane in (+)-limonene resistance

For this study we used an *E. coli* MC4100 Δ*lptD4213* strain. This mutation disrupts the outer membrane permeability barrier, making *E. coli* sensitive to antimicrobial compounds that are not normally effective against Gram-negative bacteria [41].

**Figure 5:**
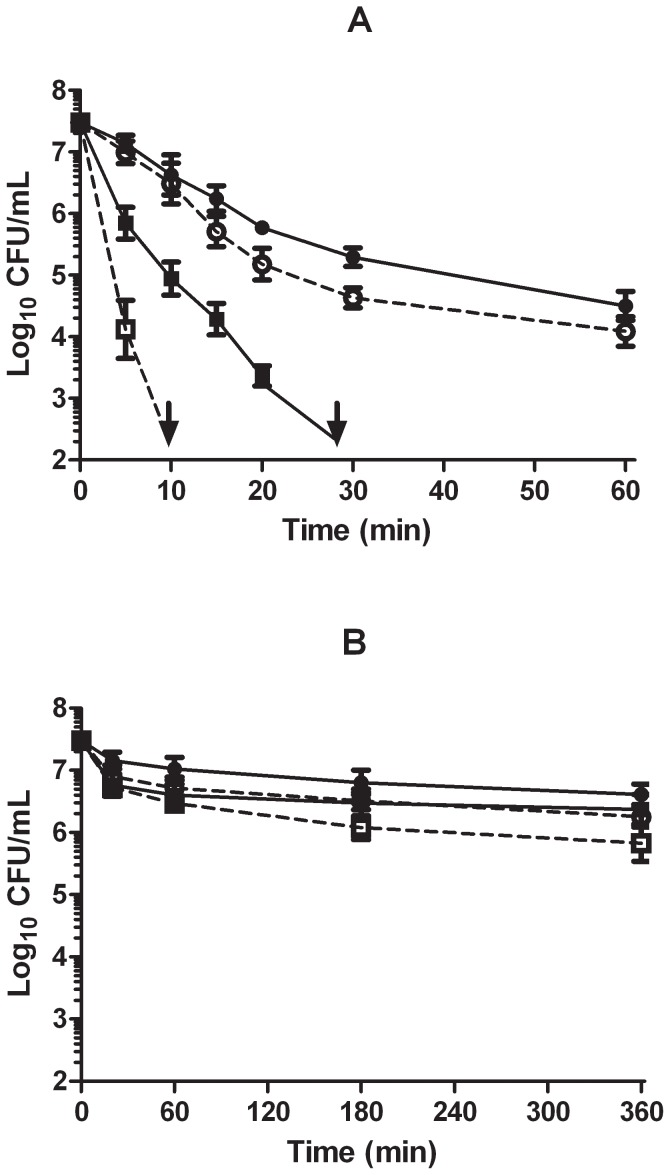
Effect of increased outer membrane permeability on *Escherichia coli* MC4100 resistance to (+)-limonene. Survival curves of *Escherichia coli* MC4100 (•) and its defective mutant Δ*lptD4213* (▪) after exposure to 200 µL/L of (+)-limonene in citrate-phosphate buffer of pH 4.0 (A) and pH 7.0 (B). Cells were recovered in TSAYE (•, ▪) and TSAYE-SC (sodium chloride: ○, □). Arrows indicate survival counts under detection limit. Error bars indicate standard error.

The *lptD4213* mutant was less resistant to (+)-limonene at pH 4.0 than its wild type strain (Figure 5A). For example, after 30 min more than 5 log_10_ cycles (>99.999%) of the initial *lptD4213* population were dead, whereas less than 2.5 log_10_ cycles (99.7%) of the wild strain population were inactivated. Surviving counts in Figure 5A indicate that (+)-limonene did not induce sub-lethal injuries in the cytoplasmic membrane of the wild type strain MC4100. However, a high proportion (>2.5 log_10_ cycles or 99.7% of survivors) of *lptD4213* cells had sub-lethal damages in their cytoplasmic membrane after (+)-limonene treatment at pH 4.0. On the contrary, the *lptD4213* mutants treated by (+)-limonene at pH 7.0 showed the same resistance as wild type cells and sub-lethal injuries in the cytoplasmic membrane were not detected (Figure 5B).

Bacterial counts in selective or non-selective media were not modified (*p*>0.05) by incubation in citrate–phosphate buffer at pH 7.0 or pH 4.0 without (+)-limonene for 60 min at 20 °C (data not shown).

### Combined preservation processes: lethal heat treatment of *E. coli* O157:H7 in presence of (+)-limonene

To study a combined process of (+)-limonene with a lethal heat treatment, a pathogenic *E. coli* serotype, *E. coli* O157:H7, and acid fruit juices as treatment medium were chosen. Preliminary results showed that *E. coli* O157:H7 (+)-limonene resistance at 20°C was similar (data not shown).

**Figure 6:**
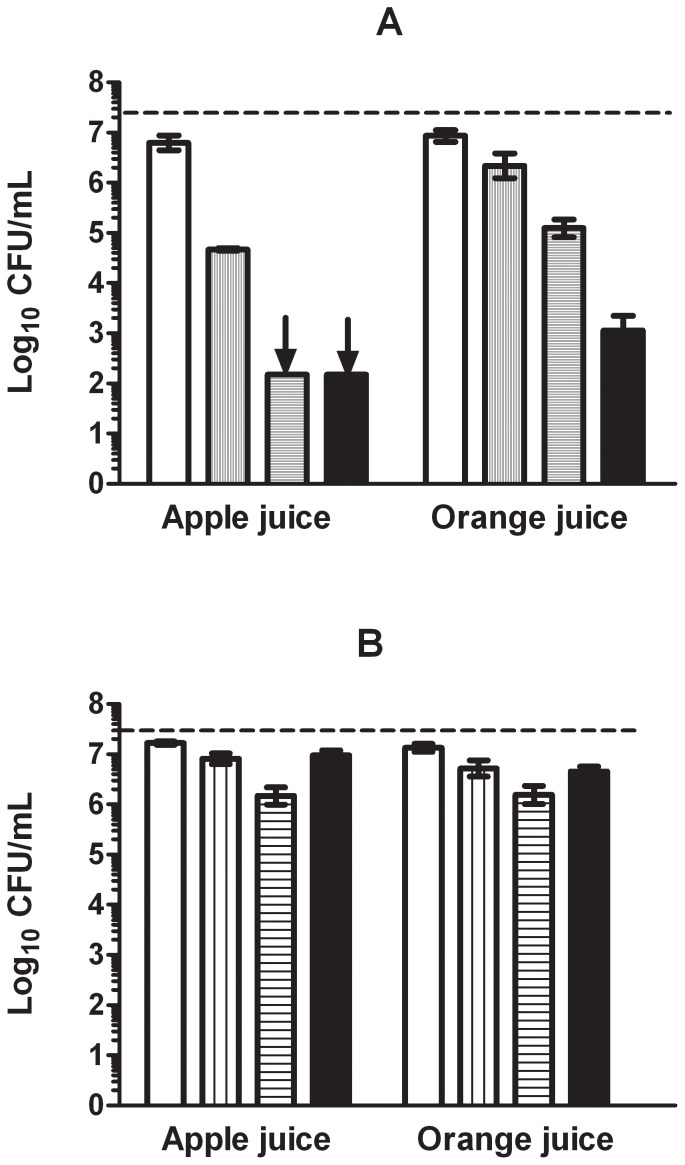
Inactivation of *Escherichia coli* O157:H7 by combined processes of heat and Pulsed Electric Treatments with (+)-limonene. Log_10_ of survival counts of *Escherichia coli* O157:H7 after lethal heat treatments (54° C for 10 min) (A) or Pulsed Electric Fields treatments (30 kV/cm and 25 pulses) (B) in absence (□) or presence of 200 µL/L of (+)-limonene (▪) and recovery onto TSAYE. Cells after heat and PEF treatments without (+)-limonene were also recovered onto TSAYE-SC (sodium chloride: vertical stripes) or TSAYE-BS (bile salts: horizontal stripes). Discontinuous line indicates initial cell concentration (3·10^7^ CFU/mL). Arrows indicate survival counts under detection limit. Error bars indicate standard error.

Figure 6A shows the inactivation of *E. coli* O157:H7 by a lethal heat treatment (54°C for 10 min) alone or in combination with 200 µL/L of (+)-limonene in apple or orange juice. A lethal heat treatment alone inactivated 0.5 log_10_ cycles of the initial population of *E. coli* O157:H7; and caused sub-lethal damages in the cytoplasmic membrane in about 2 and 0.5 log_10_ cycles of survivors (as seen by the difference in log_10_ counts between recovery in TSAYE and TSAYE with sodium chloride) when cells were treated in apple and orange juice, respectively. Moreover, 4.5 and 2 log_10_ cycles of survivors showed sub-lethal damages in their outer membrane (as seen by the difference in log_10_ counts between recovery in TSAYE and TSAYE with bile salts) after lethal heat treatments in apple or orange juice, respectively.

The combined process of lethal heat and (+)-limonene in both juices caused the inactivation of more than 4 extra log_10_ cycles as compared with application of separate treatments. Hence, this combination in juices resulted in a synergistic effect on the final inactivation. A synergistic effect between (+)-limonene and lethal heat treatments under the same treatment conditions was also observed in citrate-phosphate buffer at pH 7.0. As observed at pH 4.0, simultaneous application of both treatments at pH 7.0 allowed the inactivation of more than 4 extra log_10_ cycles (data not shown).

Bacterial counts in selective or non-selective media were not modified (*p*>0.05) by incubation in apple or orange juice without (+)-limonene for 60 min at 20 °C (data not shown).

### Combined preservation processes: Pulsed Electric Fields treatment of *E. coli* O157:H7 combined with (+)-limonene

Figure 6B shows the inactivation of *E. coli* O157:H7 by a mild PEF treatment (25 pulses at 30 kV/cm) alone or in combination with 200 µL/L of (+)-limonene in apple or orange juice.

On the one hand, a separate treatment of 200 µL/L (+)-limonene at 20°C against 3·10^7^ CFU/mL of *E. coli* O157:H7 when suspended in these juices for 10 min inactivated less than 0.5 log_10_ cycles of the initial population (data not shown). On the other hand, a separate PEF treatment in absence of (+)-limonene inactivated less than 0.5 log_10_ cycles of the initial *E. coli* O157:H7 population, and caused sub-lethal injury in the outer membrane in less than 1 log_10_ cycle of surviving cells.

The final level of inactivation resulting from the combined process (PEF with (+)-limonene) was additive, i.e. was equal to the sum of the levels of inactivation of both treatments applied separately, not observing any extra inactivation because of the simultaneous application of a lethal heat treatment in presence of (+)-limonene.

Bacterial counts in selective or non-selective media were not modified (*p*>0.05) by incubation in apple or orange juice without (+)-limonene for 60 min at 20 °C (data not shown).

## Discussion

Previous research on the antibacterial activity of (+)-limonene has been mostly focused on its bacteriostatic activity [7], [42], but little is known about its activity as a bactericidal agent in food preservation. In this respect, an important aspect to consider is the pH of the treatment medium (or the food matrix), since the final inactivation achieved by (+)-limonene was considerably higher in acid conditions (Figure 1). It is generally considered that the bacterial resistance to essential oils (EO) and their components decreases with lowering pH values because of the increase in EO hydrophobicity at low pH. As a consequence, there is an easier EO dissolution in the lipids of the cell membrane [43].

Our research was divided into two well-differentiated parts. The first part is focused on the study of mechanism of bacterial inactivation by (+)-limonene for which two wild-type and mutant strains (BJ4 and its *rpoS* mutant, and MC4100 and its *lptD4213* mutant) were used. The second part of our study is dedicated to a practical application in fruit juices of the knowledge obtained in the first part in order to demonstrate the key role of the outer membrane in microbial protection against (+)-limonene. For this objective, *E. coli* O157:H7 was used owing to its importance in food safety of fruit juices.

The expression of RpoS has been reported to cause physiological and morphological modifications that increase microbial resistance to various stresses [44]. Since deletion of *rpoS* did not decrease *E. coli* BJ4 resistance to (+)-limonene (Figure 1), probably the expression of σ^S^-controlled genes under stationary-phase conditions, such as *dps* (a stress response DNA-binding protein) or *uspB* (universal stress protein B) [19], [20], [21] did not play a role in cell resistance to (+)-limonene. This finding would suggest different mechanisms of inactivation and microbial resistance for (+)-limonene in relation to other antimicrobial compounds such as citral [13] and food preservation technologies [44].

The application of a sub-lethal heat, cold or acid shock has been demonstrated to induce cross resistance to multiple stresses (see review [45]) in *E. coli*. In this study, we have shown that a previous sub-lethal heat or acid shock did not influence subsequent *E. coli* BJ4 resistance to (+)-limonene (Table 1). Interestingly, *rpoS* deletion did not modify (+)-limonene resistance of sub-lethally heat- and acid-shocked cells. However, a previous sub-lethal cold-shock decreased the resistance of both wild type and *rpoS* mutant cells to (+)-limonene (Table 1). Exposure to cold temperatures leads to a decrease in the membrane fluidity which triggers an increase in the ratio of unsaturated fatty acids [46], [47], that could be responsible of the decreased resistance to (+)-limonene (Table 1).

The occurrence of sub-lethal injuries after food preservation treatments can be evaluated using different techniques, such as different survival counts obtained between plating treated cells in non-selective and selective media [48], and delay in lag phase before starting growth in treated with regards non-treated cells [49], [50]. At the conditions assayed in this study no sub-lethal damage in the cell envelopes was detected after exposure of *E. coli* BJ4 to (+)-limonene by the selective media plating technique (Figure 2). Furthermore, the same duration of lag phase was observed for treated and untreated *E. coli* BJ4 cells, suggesting that neither the cell envelopes nor other cell structures were sub-lethally injured. Therefore, the action of (+)-limonene could be catalogued under the “quantal” effect, a response which can be expressed in binary terms: it is either present or absent (“all or nothing”) [51] in which bacteria are either killed or intact after the treatment. Occurrence of sub-lethal damage in *E. coli* BJ4 cell envelopes by other EO compounds, such as citral or carvacrol [13], [14], would suggest a different mechanism of inactivation between these compounds and (+)-limonene.

Food preservation technologies, such as heat, pulsed electric fields (PEF), high hydrostatic pressure (HHP) and essential oils (EOs) normally target cell envelopes [52], [53], [54]. Thus, we evaluated membrane permeabilization in *E. coli* BJ4 using propidium iodide. A direct correlation between the percentage of cell inactivation and the percentage of membrane permeabilization was obtained (Figure 3). The simultaneous occurrence of both phenomena identifies the cell envelopes as an important target in the mechanism of *E. coli* BJ4 inactivation by (+)-limonene.

To further study the damages caused by (+)-limonene, we included an analysis by ATR-IRMS. We used this technique that evaluates the biochemical composition of the bacterial cell constituents [16], such as water, proteins, nucleic acids, fatty acids and polysaccharides, to describe the changes caused by (+)-limonene. ATR-IRMS results allowed selecting two major discriminating bands at both pH 4.0 (Figure 4C) and pH 7.0 (Figure 4F) as the main responsible for the differences between untreated and (+)-limonene-treated *E. coli* BJ4 cells. At pH 4.0, the 1,624 cm^−1^ band corresponding to the amide I absorption band of β-sheet proteins [35], [36]; and the band at 1,395 cm^−1^ reflecting the symmetric stretching of COO^-^ groups in amino acids and/or fatty acids [37], [38], [39]. Since β-barrel membrane proteins occur in the outer membranes of Gram-negative bacteria [55], the main contribution to the discrimination between untreated and (+)-limonene-treated cells at pH 4.0 could come from affected outer membrane proteins that form membrane-spanning β-barrels. However, at pH 7.0, the discriminating bands for (+)-limonene treatments were found at 1,083, 1,250 and 992 cm^−1^, corresponding to the symmetric (1,083 cm^−1^) and asymmetric (1,250 cm^−1^) stretching of P = O groups in phosphodiester bonds and ring vibrations of carbohydrates (992 cm^−1^) [16], [37], [40]. Phosphodiester bonds are present in phospholipids of the cytoplasmic membrane and of the inner leaflet of the outer membrane [56], while carbohydrates are found in the lipopolysaccharide (LPS) fraction of the cell wall [57]. In consequence, (+)-limonene would target phospholipids and LPS cell fraction at pH 7.0, and the protein fraction at pH 4.0 in *E. coli* BJ4.

It should be noted that these conclusions related to the mechanism of bacterial inactivation by (+)-limonene were drawn from experiments using the Gram-negative strains *E. coli* BJ4 and its Δ*rpoS* mutant. Further experiments using different microorganisms are needed to extrapolate these conclusions to other Gram-negative strains.

Once we confirmed the role of cell envelopes in the (+)-limonene antimicrobial activity, we used the mutant strain Δ*lptD4213* (formerly known as Δ*imp4213*) to evaluate the role of outer membrane in the mechanism of inactivation by (+)-limonene (Figure 5). LptD is an essential β-barrel protein of the outer membrane [58] which is implicated in lipopolysaccharide (LPS) assembly [59]. Depletion of this protein results in increased outer membrane permeability to lipophilic compounds, such as novobiocin or rifampin [24]. In effect, at pH 4.0 *lptD4213* mutants showed a decreased (+)-limonene resistance, and occurrence of sub-lethal damage in the cytoplasmic membrane was demonstrated after (+)-limonene treatments. This finding, together with ATR-IRMS observations, could indicate that, at pH 4.0, (+)-limonene should damage the outer membrane in order to gain access to the periplasmic space and cytoplasmic membrane and inactivate the bacterial cell. Once outer membrane permeability to (+)-limonene is increased, there would be an enhanced interaction of (+)-limonene molecules at pH 4.0 with the components in the cytoplasmic membrane. However, the bactericidal action of (+)-limonene at pH 7.0 was not enhanced by higher outer membrane permeability in *lptD4213* mutants, indicating that facilitation of (+)-limonene access to the periplasmic space and cytoplasmic membrane would not be required at pH 7.0. Furthermore, results shown by ATR-IRMS would indicate that LPS damage was related to mechanism of inactivation by (+)-limonene at pH 7.0. Therefore, mechanism of *E. coli* BJ4 and MC4100 inactivation by (+)-limonene was different as a function of the pH of the treatment medium. In spite of differences between *E. coli* BJ4 and MC4100, (+)-limonene resistance shown by both strains was similar (Figures 1 and 5). However, further research using other microorganisms is needed in order to increase the knowledge on the mechanism of bacterial inactivation by (+)-limonene and to use this compound in practical applications.

From the study of the mechanism of inactivation by (+)-limonene in *E. coli* BJ4 and MC4100 we could expect that application of a food preservation technology causing sub-lethal damages in outer membrane would increase the lethal effect induced by (+)-limonene, leading to an advantageous combined process. In order to prove this hypothesis and to provide a practical application of this knowledge we studied the effect of (+)-limonene in a combined process with heat or PEF in *E. coli* O157:H7 because of the presence of an outer membrane in this pathogenic serotype and its importance in food safety of fruit juices [60], [61]. We determined that combinations of a lethal heat treatment that damaged outer membrane with (+)-limonene also achieved a synergistic effect to inactivate *E. coli* O157:H7 in juice. Thus, a facilitated access of (+)-limonene to the periplasmic space and cytoplasmic membrane would cause the inactivation of these sub-lethally damaged cells (Fig. 6A). On the contrary, since PEF did not cause sub-lethal damage to the outer membrane of *E. coli* O157:H7 the combination of (+)-limonene and PEF did not yield any extra inactivation when compared to the inactivation by PEF alone at the assayed conditions (Figure 6B). Since *E. coli* O157:H7 is a virulent strain whose genome has a significant number of differences from other *E. coli* strains, such as the presence of more than 1,300 new genes [62], [63], transfer of the knowledge on mechanism of microbial inactivation by (+)-limonene from *E. coli* BJ4 and MC4100 to *E. coli* O157:H7 would require further studies on the influence of the factors investigated in this research.

Although preliminary results indicate that (+)-limonene concentrations used in this study were accepted by consumers, sensory analysis of apple juice with (+)-limonene should be performed to evaluate commercial viability. Previous work with citral and PEF in *E. coli* BJ4 reached a similar conclusion [13], as well as combined processes between PEF and different antimicrobials against *E. coli* O157:H7 in apple and orange juices [14].

## Conclusion

The study of the mechanism of bacterial inactivation by (+)-limonene showed that the lethality of this compound was higher at pH 4.0 than at neutral pH. Contrary to other food preservation treatments, deletion of *rpoS* did not modify *E. coli* BJ4 resistance to (+)-limonene. Furthermore, a previous sub-lethal heat or acid shock did not change *E. coli* BJ4 resistance to (+)-limonene, independently of *rpoS* deletion. However, a previous sub-lethal cold shock decreased the resistance of wild-type *E. coli* BJ4 and even more the resistance of *rpoS* mutant to (+)-limonene. Assessment of *E. coli* BJ4 permeabilization with propidium iodide showed that this phenomenon occurred simultaneously with bacterial inactivation, identifying the cell envelopes as important (+)-limonene targets. In contrast to other essential oils compounds, (+)-limonene did not cause sub-lethal injuries in any *E. coli* BJ4 structure, cataloguing its lethal action under the “quantal” effect (“all or nothing”). Different resistance pattern of *lptD4213* mutants and ATR-IRMS results showed the importance of outer membrane in the mechanism of inactivation by (+)-limonene at pH 4.0. At pH 7.0, increased outer membrane permeability did not lead to a decreased (+)-limonene resistance and ATR-IRMS spectra demonstrated the importance of LPS in the mechanism of *E. coli* BJ4 inactivation at this pH. Considering the orange-like flavor of (+)-limonene and its consideration as a GRAS (Generally Recognized As Safe) substance [64], [65], we propose the simultaneous application of (+)-limonene with other preservation technologies that damage outer membrane, such as heat treatments, in order to design combined food preservation processes with a synergistic lethal effect, as demonstrated for *E. coli* O157:H7 in this study. Although bacterial resistance of the studied *E. coli* strains was similar, further research is needed in order to increase the knowledge on the mechanism of inactivation by (+)-limonene in other bacteria and to use this compound in practical applications.
